# The Potential Role of the Early Maladaptive Schema in Behavioral Addictions Among Late Adolescents and Young Adults

**DOI:** 10.3389/fpsyg.2019.03022

**Published:** 2020-01-21

**Authors:** Matteo Aloi, Valeria Verrastro, Marianna Rania, Raffaella Sacco, Fernando Fernández-Aranda, Susana Jiménez-Murcia, Pasquale De Fazio, Cristina Segura-Garcia

**Affiliations:** ^1^Department of Health Sciences, University “Magna Graecia” of Catanzaro, Catanzaro, Italy; ^2^Department of Medical and Surgical Sciences, University “Magna Graecia” of Catanzaro, Catanzaro, Italy; ^3^Department of Psychiatry, Bellvitge University Hospital, IDIBELL, Barcelona, Spain; ^4^CIBER Fisiopatología de la Obesidad y Nutrición, Instituto de Salud Carlos III, Barcelona, Spain; ^5^Department of Clinical Sciences, School of Medicine and Health Sciences, University of Barcelona, Barcelona, Spain

**Keywords:** behavioral addiction, early maladaptive schemas, food addiction, internet addiction, gambling disorder, adolescents

## Abstract

**Background:**

Behavioral addiction (BA) is a recent concept in psychiatry. Few studies have investigated the relationship between BA and early maladaptive schemas (EMSs). EMS is the core of Schema Therapy (ST). According to the ST model, psychiatric disorders result from the development of EMSs in response to unmet emotional needs in childhood. Bach et al. (2018) grouped the 18 EMSs into four domains: (1) *disconnection and rejection*; (2) *impaired autonomy and performance*; (3) *excessive responsibility and standards*; and (4) *impaired limits*. This study aims to assess the possible association of the most frequent BAs with EMSs in a large group of late adolescents and young adults and to evaluate their self-perceived quality of life (QoL).

**Methods:**

A battery of psychological tests assessing food addiction (FA), gambling disorder (GD), internet addiction (IA), and QoL was administered to 1,075 late adolescents and young adults (*N* = 637; 59.3% women). A forward-stepwise logistic regression model was run to identify which variables were associated with BAs.

**Results:**

Food addiction was more frequent among women and GD among men, while IA was equally distributed. Regarding the EMSs, participants with FA or IA showed significantly higher scores on all four-schema domains, whereas those with GD exhibited higher scores on *impaired autonomy and performance* and *impaired limits*. Besides, average scores of all domains increased with the association of two or more comorbid BAs. Self-perceived QoL was lower for participants with FA and IA, but not for those with GD; the presence of comorbid BAs was associated with lower Physical Component Summary (PCS) and Mental Component Summary (MCS) scores. Finally, specific EMS domains and demographic variables were associated with each BA.

**Conclusion:**

Late adolescents and young adults with FA or IA have a lower perception of their mental and physical health. The most striking result is that FA appears to be associated with the *disconnection and rejection* schema domain, IA with all the schema domains (except for *impaired autonomy and performance*), and GD with *impaired autonomy and performance* schema domain. In conclusion, our findings suggest that EMS should be systematically assessed during psychotherapy of patients with BAs.

## Introduction

The construct of behavioral addictions (BAs) is a recent concept in psychiatry. In fact, it was first included in 2013 in the fifth edition of the Diagnostic and Statistical Manual of Mental Disorders (DSM-5) as an official psychiatric diagnosis. So, in clinical practice, it is necessary to distinguish between substance and BAs. In fact, even if BAs do not appear in the DSM-5, except for gambling disorder (GD) and Internet gaming disorder (IGD, which is included in Section III among the “Conditions for Further Study”), they share the following characteristics with substances addiction: the age of onset in adolescence and early adulthood, the specific features (i.e., impaired control, functional impairment, and persisting engagement in the behavior despite negative consequences), and the clinical course (Sussman et al., 2017).

Other problematic behaviors, such as food addiction (FA) and Internet addiction (IA), have aroused interest in recent years (Jiménez-Murcia et al., 2017; Romero et al., 2019). Although there are still insufficient data to allocate these disorders into the diagnostic category of psychiatric disorders, they seem to share many clinical characteristics with BAs (Meule and Gearhardt, 2014; Petry et al., 2018). The notion of FA theorizes that individuals experience addictive-like symptoms related to the consumption of high calorie/palatable foods, which leads to clinically significant impairment or distress on several areas of functioning (Gearhardt et al., 2011). Recently, researchers in the field of eating disorders (EDs) have claimed that FA should be comprised within the spectrum of EDs (Wiss and Brewerton, 2017; Fernandez-Aranda et al., 2018; Jiménez-Murcia et al., 2019); on the contrary, others believe that FA does not yet have enough evidence to be an independent nosographic category (Hebebrand et al., 2014). Regarding IA, it may be considered an autonomous nosographic entity from IGD given that the problematic or excessive use of the Internet is not necessarily related to gaming for many people (Griffiths and Pontes, 2014). Recent findings demonstrate that even if IGD and IA can share online gaming, distinctive characteristics of IA are online chatting and social networking, while male gender is distinctive of IGD (Király et al., 2014). Consequently, data seem to support the notion that IA and IGD are distinct nosological entities.

All of these addictions, however, are associated with a pattern of emotional dysregulation and cognitive distortions, which are typical of behaviors that people use to seek immediate gratification. It has been hypothesized that dysfunctional behavior could be a maladaptive strategy for dealing with negative emotions and feelings, such as frustration, inadequacy, and isolation (Zeeck et al., 2010). Furthermore, many studies have reported poor quality of life (QoL) among people with BAs because they are associated with physical comorbidities (i.e., withdrawal symptoms), social problems (i.e., isolation and social withdrawal, problems at work or at home), psychiatric disorders (i.e., depression, anxiety), and lower life satisfaction (Hoseinifar et al., 2011; Lu et al., 2018; Wang et al., 2019). For this reason, behavioral addicts need much help and support. Besides, gender seem to moderate the link between BAs and their clinical phenotype. In fact, recent findings showed that while GD was more frequent among males than females (Fröberg et al., 2015; Di Nicola et al., 2017; Carneiro et al., 2019), IA did not show a gender-related trend (McNicol and Thorsteinsson, 2017; Li et al., 2019) and FA was more frequent among females (Aloi et al., 2017; Borisenkov et al., 2018; Magyar et al., 2018).

Perhaps the most relevant point to highlight is that research has mainly focused on the presence/absence of symptoms of BAs, whereas the etiological processes and peculiar characteristics of BAs have rarely been investigated. Moreover, some of these symptoms, such as withdrawal and tolerance, are difficult to apply and measure in relation to behaviors (Starcevic, 2016; Kardefelt-Winther et al., 2017).

According to the theoretical model of Schema Therapy (ST), psychiatric disorders might result from the formation of early maladaptive schemas (EMSs) in response to unmet needs in childhood. EMSs are defined as “extremely stable and enduring themes, comprised of memories, emotions, cognitions, and bodily sensations regarding oneself and one’s relationship with others, that develop during childhood and are elaborated on throughout the individual’s lifetime, and that are dysfunctional to a significant degree” (Young et al., 2003). The new proposed model (Bach et al., 2018) grouped 18 EMSs into four specific domains: (1) *disconnection and rejection*: All the schemas of this domain are associated with a lack of security, safety, and stability in interpersonal relationships; (2) *impaired autonomy and performance*: People with schemas of this domain perceive themselves as insecure and suffer from a lack of autonomy, i.e., they are afraid that autonomous decisions may damage important relationships, or they expect to fail in situations where they must provide a performance; (3) *excessive responsibility and standards*: People with patterns in this domain typically put the needs, desires and wishes of others before their own, and, as a result, many of their efforts are directed toward satisfying the wishes of others; and (4) *impaired limits*: A lack of internal limits, an inability to form long-term goals, and a lack of responsibility to others.

Many studies have found that EMSs are associated with the development of personality disorders (Sempértegui et al., 2013) and other psychopathological conditions (Basile et al., 2017; Aloi et al., 2019; Rania et al., 2019). EMSs have also recently been investigated in samples with GD (Shorey et al., 2012) and substance-use disorders (SUDs) (Shorey et al., 2013, 2014), but only a few studies have investigated the relationship between BAs and EMSs (Elmquist et al., 2016; Shajari et al., 2016; Imperatori et al., 2017). From the literature, it appears that GD patients are characterized by numerous EMSs, in particular by the schema domain *impaired autonomy and performance* (Shorey et al., 2012). IA seems to be associated with all the schema domains (Shajari et al., 2016). Finally, researchers found that the *disconnection/rejection* schema domain was independently associated with FA severity, suggesting that this domain may be a crucial factor for the development of FA (Imperatori et al., 2017).

Based on the above, this study has two aims:

(1) To assess the possible association of specific BAs (i.e., FA, IA, and GD) with EMSs and gender in a large group of late adolescents and young adults. More in detail, according to the previous research, we expect to find: FA associated with the *disconnection and rejection* schema domain; IA associated with all the schema domains; and, GD associated with *impaired autonomy and performance* schema domain. Regarding the possible role of gender in the development of BAs, we suppose to find the male gender associated with GD, the female gender with FA, and no gender difference on IA.
(2) To evaluate their self-perceived QoL; in particular, we expect to find a worse QoL among participants with any BAs.

## Materials and Methods

### Participants and Procedure

First- and third-year university students and senior high school students from the same catchment area in southern Italy participated in the research.

Through an anonymous online survey, the participants completed an informed consent form and the questionnaires, and they also provided their heights and weights. Answers of participants who did not complete all items were dropped from the electronic database automatically. Anonymity was guaranteed through the use of nicknames (formed by at least eight alphanumeric and symbol characters). No grant or economic compensation was offered to participants.

The final sample consisted of 1,075 participants (*N* = 637; 59.3% women) with a mean age of 19.69 ± 1.7 (17–24) years old; 810 (80.5%) participants had middle school diplomas while 210 (19.5%) had high school diplomas. All participants were Caucasian. The present study was carried out in accordance with the latest version of the Declaration of Helsinki and was approved by the local Ethical Committee. All participants provided informed consent before participating and parental consent was sought for those younger than 18 years of age.

### Measures

#### Young Schema Questionnaire Short Form-3

The Young Schema Questionnaire Short Form-3 (YSQ-S3) is a self-report instrument that consists of 90 items rated on a six-point scale (from “completely untrue of me” to “describes me perfectly”) that accounts for 18 EMSs (Young, 2005). Different validations of YSQ-S3 in various languages suggest that the YSQ-S3 is a sound instrument for measuring schemas, including factorial validity and test–retest stability as well as convergent and discriminant validity.

These EMSs are grouped together into four domains: *disconnection and rejection*, *excessive responsibility and standards*, *impaired autonomy and performance*, and *impaired limits* (Bach et al., 2018). Internal consistency of the four domains were: *disconnection and rejection* 0.919, *impaired autonomy and performance* 0.904, *excessive responsibility and standards* 0.791, and *impaired limits* 0.834, indicating very good reliability.

#### Internet Addiction Test

The Internet Addiction Test (IAT) is a self-report test that assesses problematic Internet use and consists of 20 items on 5-point Likert-type (Young, 1998; Servidio, 2017). The total scores range from 20 to 100. According to Young’s criteria, total IAT scores of from 20 to 49 denote an average internet user, who can sometimes use the web a little too long without losing control of the situation; scores 50–79 denote over-users with frequent problems due to their Internet usage; scores 80–100 denote a use of the Internet that is very intense and causes significant problems for the person. In the current study, we used the cut-off 80–100 to diagnose IA. Cronbach’s alpha in this study was 0.879.

#### South Oaks Gambling Screen

South Oaks Gambling Screen (SOGS) is the most widely known and used questionnaire for the screening of GDs and is composed of 16 questions, including 37 items, which ask the subjects about their gambling activity and associated behaviors throughout their lifetime. There are 20 scoring items, all equally weighed, requiring a “yes” or “no” answer, so scores range from 0 to 20 (Lesieur and Blume, 1987; Barbaranelli et al., 2013). Scores 3 to 4 indicate at-risk gambling, scores ≥5 indicate problematic gambling and ≥9 indicate a serious GD. In this study, we used the cut-off SOGS ≥9 to diagnose GD. Cronbach’s alpha in the present research was 0.848.

#### Yale Food Addiction Scale 2.0

The Yale Food Addiction Scale 2.0 (YFAS 2.0) evaluates addiction-like eating behavior over the preceding 12 months (Gearhardt et al., 2016; Aloi et al., 2017). It consists of 35 items, which are scored on an eight-point scale ranging from never (score = 0) to every day (score = 7) to assess symptoms related to the 11 diagnostic criteria for substance-related and addictive disorders (SRAD) of DSM-5. The 11 symptoms of FA following the DSM-5 criteria of SRAD are: overeating (Criterion 1), desire to cut down (Criterion 2), time spent (Criterion 3), craving (Criterion 4), related impairment (work/school, family, social relationship) (Criteria 5–7), risky use (physically hazardous, detrimental physical/psychological consequences) (Criteria 8–9), tolerance (Criterion 10), and withdrawal (Criterion 11).

Each of the 11 diagnostic criteria was considered present if one or more of the relevant questions for each criterion reached the threshold. For the specific threshold of each item, it is possible recover them in the validation of the scale^1^ Two types of scoring are possible: (1) a final symptom-count score can be calculated by adding up all of the endorsed symptoms; (2) the severity level is described according to the diagnostic thresholds for SRAD in DSM-5: mild FA (2–3 symptoms), moderate FA (4–5 symptoms), and severe FA (≥6 symptoms). Finally, every FA “diagnosis” requires the presence of the distress or impairment criteria. In this study, we used the cut-off≥6 symptoms (severe FA) to diagnose FA. Kuder–Richardson’s alpha for dichotomous variables was run and in this study was 0.880.

#### Short Form-12 Health Survey

The Short Form-12 Health Survey (SF-12) is a widely used instrument for assessing QoL and consists of 12 items derived from the SF-36 (Ware et al., 1996; Apolone et al., 2001). These items are grouped into two domains, named Physical Component Summary (PCS) and Mental Component Summary (MCS), both including six items. Responses to questions are dichotomous (yes/no), ordinal (excellent to poor), or expressed by a frequency (always to never). The total score ranges from 0 to 100, with higher scores referring to higher QoL. In this study, Cronbach’s alpha coefficient for the PCS-12 and MCS-12 scores were 0.84 and 0.81, respectively.

### Statistical Analysis

Data analyses were performed using the Statistical Package for the Social Sciences (SPSS) version 21.0 (SPSS 21.0; SPSS Inc., Chicago, IL, United States). Descriptive statistics included frequencies and percentages, and means and standard deviations, as appropriate. Differences between groups were assessed with chi-squared for categorical variables and *t*-tests or ANOVA for continuous data, as appropriate. Additionally, Cohen’s *d* or η^2^ were, respectively, calculated for significant results in *t*-tests or ANOVA as measures of effect size (ES). Values of 0.2, 0.6, 1.2, and >1.2 can be, respectively, categorized into slight, small, moderate, and large ES for Cohen’s *d* (Cohen, 1988). In the case of η^2^ values of 0.01, 0.06, and 0.14 indicate small, medium, and large ES (Levine and Hullett, 2002). A forward-stepwise logistic regression model was run to identify which variables were associated with BAs. In all cases, differences were considered to be significant when *p* < 0.05.

## Results

Table 1 describes the differences between males and females. Average scores of questionnaires assessing dysfunctional behaviors are as follows: YFAS total present criteria = 1.25 ± 2.3 (0–11); SOGS = 0.6 ± 1.7 (0–16); IAT = 40.1 ± 11.4 (20–85). Overall, 88 (8.2%) participants were positive to YFAS 2.0, 184 (17.1%) to IAT, and 73 (6.8%) to SOGS; besides, among these, 50 participants were positive to two tests (20 IAT/YFAS, 20 IAT/SOGS, 10 SOGS/YFAS) and only 3 participants were simultaneously positive to all measures. FA was more frequent among women, and GD was more frequent among men, while IA was equally distributed (Table 1).

**TABLE 1:** Sample demographics and distribution of behavioral addictions.

		**Females**		**Males**		**Statistics**	***p***	***d*/φ^c^**
Age^a^		18.8	(1.7)	18.6	(1.7)	*t* = 1.491	0.136	
BMI^a^		21.6	(3.6)	23.2	(3.7)	*t* = −7.389	<0.001	0.44
YFAS 2.0^b^	Negative	576	(90.4)	411	(93.8)	χ^2^ = 4.020	0.045	0.06
	Positive	61	(9.6)	27	(6.2)			
SOGS^b^	Negative	625	(98.2)	377	(86.1)	χ^2^ = 59.471	<0.001	0.24
	Positive	12	(1.8)	61	(13.9)			
IAT^b^	Negative	533	(83.7)	358	(81.7)	χ^2^ = 0.687	0.407	
	Positive	104	(16.3)	80	(18.3)			

*^*a*^Results are presented as means (SD). ^*b*^Results are presented as frequencies (%). BMI, Body Mass Index; YFAS 2.0, Yale Food Addiction Scale 2.0; SOGS, South Oaks Gambling Screen; IAT, Internet Addiction Test. ^*c*^Only effect sizes of significant differences are displayed. Cohen’s *d* for continuous variables and φ for categorical variables.*

Table 2 summarizes the results of the comparison between BAs and YSQ-S3 domains. Participants who had positive scores with the YFAS 2.0 and the IAT showed significantly higher scores on all four-schema domains, whereas participants who were positive on the SOGS exhibited higher scores on the *impaired autonomy and performance* and *impaired limits* schema domains. As displayed on the last row of the table, average scores of all domains increased with the association of two or more comorbid BAs.

**TABLE 2:** Comparison between behavioral addictions and YSQ-S3 domains.

		**Disconnection and**		**Impaired autonomy**		**Excessive responsibility**		**Impaired**	
		**Rejection**		**and performance**		**and standards**		**limits**	
YFAS 2.0^a^	Negative	2.1	(0.9)	1.8	(0.7)	2.8	(0.9)	2.4	(0.9)
	Positive	3.3	(1.1)	2.7	(1.0)	3.5	(0.9)	3.1	(0.9)
	*t*	−11.414		−9.933		−6.743		−6.866	
	*p*	<0.001		<0.001		<0.001		<0.001	
	*d*^b^	1.19		1.04		0.78		0.78	
SOGS^a^	Negative	2.2	(1.0)	1.9	(0.8)	2.8	(0.9)	2.4	(0.9)
	Positive	2.4	(1.2)	2.2	(1.1)	2.9	(1.2)	2.8	(1.1)
	*t*	−1.806		−3.491		−0.547		−3.258	
	*p*	0.071		0.001		0.584		0.001	
	*d*^b^			0.31				0.40	
IAT^a^	Negative	2.1	(0.9)	1.8	(0.8)	2.8	(0.9)	2.3	(0.9)
	Positive	2.7	(1.1)	2.3	(0.9)	3.0	(1.0)	2.9	(1.0)
	*t*	−7.786		−8.142		−2.836		−7.172	
	*p*	<0.001		<0.001		0.005		0.001	
	*d*^b^	0.60		0.59		0.21		0.63	
BA comorbidity	Negative	2.1	(0.9)	1.8	(0.7)	2.8	(0.9)	2.3	(0.9)
	Positive 1 BA	2.6	(1.1)	2.3	(0.9)	3.0	(1.0)	2.8	(1.0)
	Positive >1 BA	3.2	(1.2)	2.7	(1.0)	3.2	(1.1)	3.1	(1.1)
	*F*	64.539		68.984		13.466		46.332	
	*p*	<0.001		<0.001		<0.001		<0.001	
	η^2^	0.107		0.114		0.025		0.080	

*^*a*^Results are presented as means (SD). ^*b*^Only effect sizes of significant differences are displayed. YSQ-S3, Young Schema Questionnaire Short Form 3; YFAS 2.0, Yale Food Addiction Scale 2.0; IAT, Internet Addiction Test; SOGS, South Oaks Gambling Screen; BA, Behavioral Addiction.*

Table 3 displays the findings of the comparison of SF-12 dimensions between participants with and without each BA. Individuals who were positive to the YFAS 2.0 and the IAT showed significantly lower scores on both PCS and MCS. No differences in self-perceived QoL was evident between participants who were positive and negative to the SOGS. The presence of comorbid BAs was associated to lower PCS and MCS scores.

**TABLE 3:** Comparison between behavioral addictions and SF-12 dimensions.

		**SF-12 PCS**		**Statistics**	***p***	**ES^b^**	**SF-12 MCS**		**Statistics**	***p***	**ES^b^**
YFAS 2.0^a^	Negative	52.5	(6.4)	*t* = 3.246	0.001	*d* = 0.34	43.2	(10.8)	*t* = 7.823	<0.001	*d* = 0.87
	Positive	50.1	(7.5)				33.8	(10.9)			
SOGS^a^	Negative	52.3	(6.4)	*t* = 0.960	0.337		42.3	(11.1)	*t* = −1.098	0.272	
	Positive	51.6	(7.7)				43.8	(10.1)			
IAT^a^	Negative	52.5	(6.3)	*t* = 2.597	0.010	*d* = 0.21	43.1	(11.0)	*t* = 4.859	<0.001	*d* = 0.39
	Positive	51.1	(7.2)				38.8	(10.8)			
BA comorbidity	Negative	52.7	(6.2)	*F* = 7.023	0.001	η^2^ = 0.013	43.9	(10.7)	*F* = 27.504	<0.001	η^2^ = 0.049
	Positive 1 BA	51.6	(6.9)				38.1	(11.2)			
	Positive >1 BA	49.6	(8.2)				37.7	(11.3)			

*^*a*^Results are presented as means (SD). ^*b*^Only effect sizes of significant differences are displayed. SF-12 PCS, Short Form-12 Health Survey Physical Component Summary; SF-12 MCS, Short Form-12 Health Survey Mental Component Summary; YFAS 2.0, Yale Food Addiction Scale 2.0; SOGS, South Oaks Gambling Screen; IAT, Internet Addiction Test; ES, Effect size; BA, Behavioral Addiction.*

The results of the logistic regression models are shown in Table 4. Female gender, higher BMI, and the *disconnection and rejection* schema domain scores were associated with YFAS 2.0 positivity. Younger age and higher scores in the *disconnection and rejection*, *excessive responsibility and standards*, and *impaired limits* schema domains were associated with IAT positivity. Finally, male gender and impaired autonomy and performance were associated with being positive to the SOGS.

**TABLE 4:** Results of logistic regression analysis.

**Dependent variable**	**Independent variables**	***B***	**Standard Error**	**Wald**	***p***	**Exp(B)**
YFAS 2.0^a^	Categorical BMI	0.118	0.026	19.898	<0.001	1.125
	YSQ-S3 Disconnection and Rejection	0.986	0.110	79.960	<0.001	2.680
	Gender	–0.668	0.268	6.218	0.013	0.513
IAT^b^	YSQ-S3 Disconnection and Rejection	0.445	1.270	7.270	0.007	1.560
	YSQ-S3 Excessive responsibility and standards	–0.698	0.154	20.441	<0.001	0.498
	YSQ-S3 Impaired limits	0.492	0.150	10.743	0.001	1.635
	Age	–0.166	0.068	5.936	0.015	0.847
SOGS^c^	YSQ-S3 Impaired autonomy and performance	0.484	0.127	14.460	<0.001	1.623
	Gender	2.199	0.325	45.724	<0.001	9.016

*YFAS 2.0, Yale Food Addiction Scale 2.0; IAT, Internet Addiction Test; SOGS, South Oaks Gambling Screen; BMI, Body Mass Index; YSQ-S3, Young Schema Questionnaire Short Form 3. ^*a*^Model 1. Dependent variable: YFAS 2.0; −2log Likelihood = 487.566, Nagelkerke *R*^2^ = 0.246. ^*b*^Model 2. Dependent variable: IAT; −2log Likelihood = 890,515; Nagelkerke *R*^2^ = 0.138. ^*c*^Model 3. Dependent variable: SOGS; −2log Likelihood = 458,896; Nagelkerke *R*^2^ = 0.171.*

## Discussion

The study aimed to assess the possible association of some BAs (i.e., FA, IA, and GD) with EMS and at evaluating their self-perceived QoL. To the best of our knowledge, this is the first study regarding the investigation of EMSs on BA in a very large sample of young people (*n* = 1075) according to the new four schema domains model proposed by Bach et al. (2018).

Thirty-two percent of participants had positive results to at least one BA. IA was the most frequent (17.1%), followed by FA (8.1%), and then GD (6.8%).

Regarding gender distribution, in our study we found a different distribution between participants who scored positively to BAs. GD was more likely to occur among males, as previous reports have indicated (Kristiansen and Jensen, 2011; Fröberg et al., 2015). The present results are in line with another Italian study that assessed GD together with other addictions among adolescents (Di Nicola et al., 2017). FA was more frequent among females. Other authors have previously found the same trend at a lower rate (5.5% for females versus 0.8% for males) in young adults without obesity or other comorbidities (Aloi et al., 2017), which agrees with most recent investigations (Pursey et al., 2014; Mies et al., 2017; Borisenkov et al., 2018; Magyar et al., 2018). On the other hand, IA did not show a gender-related trend, with similar frequency results among males and females. Another recent cross-cultural study of problematic Internet usage in Europe described significantly higher scores in women (26%) than among men (22%). This higher trend for women was explained with the assumption that women are more prone to use online activities and social networks (Laconi et al., 2018). Nevertheless, data regarding the Italian sample showed similar rates of IA between males and females as in the present study.

Participants who were screened positive to BAs expressed significantly higher values in their schema-domain scores. All four schema-domains results were more expressed in participants who were positive to FA and IA. Instead, a more specific pattern in maladaptive schemas emerged in people who were affected by GD. *Impaired autonomy and performance and impaired limits* were the schemas that had significantly higher scores among adolescents and young adults with gambling addictions.

According to previous studies that have investigated the association between EMSs and eating psychopathology (Pugh, 2015; Imperatori et al., 2017), our findings showed that participants with FA had higher scores in all four schema domains of the YSQ-S3. Additionally, in our sample, the scores of the *impaired limits* domain were higher in the group of participants who were positive to all three of the investigated BAs. This is consistent with Young and Klosko’s (1993) study, which considered the impaired limits domain to be the most closely related to externalizing behavioral problems, such as BAs. We found an association between IA and all EMS domains, as did Shajari et al. (2016). All the above mention gains of interest as the association of more than one BA resulted in higher EMS domains.

Regarding QoL, we found that participants with IAs and FAs had low scores in physical and mental health, which was unlike those with high GD scores.

Our data corroborate the results of previous research about impaired QoL among IA subjects (Fatehi et al., 2016; Karacic and Oreskovic, 2017). IA impairs physical functions as it implies neglecting usual, daily life functioning and the acquisition of some other behaviors that can lead to physical consequences.

Literature on QoL reports poorer values in all dimensions both among normal-weight and obese adolescents with FA (Tompkins et al., 2017; Nunes-Neto et al., 2018; Wiedemann et al., 2018; Zhao et al., 2018). There is no controversy in the association between FA and the impaired physical and psychological domains, even if previous studies used different instruments to assess QoL dimensions, reporting much more discriminant subdimensions, such as emotional, school, and social functioning (Nunes-Neto et al., 2018; Zhao et al., 2018). We only took into consideration the dimensions of PCS and MCS, so we can only confirm poorer levels in these dimensions.

Quality of life was not found to be impaired among pathological gamblers. On the contrary, other studies found a stronger association with poorer mental health among gamblers than among non-gamblers (Castrén et al., 2013; Ekholm et al., 2018). Our explanation could be the different age among gambling addicts, in fact, somehow, younger gamblers tend to still be in the honeymoon phase of this addiction, while older gamblers have already developed the long-lasting side-effects with the negative physical and psychological consequences. Another explanation of this result could be found in Jiménez-Murcia et al. (2013) study. These authors clustered young pathological gamblers and concluded that three different clusters exist, with differences in terms of gambling severity, and personality and psychopathological measures. According to this clustering, it is possible that the present sample fits with the “High General Functioning (Type I)” phenotype that is characterized by a lower severity of the disorder, general psychopathology, and healthier personality traits. On the other hand, Blaszczynski and Nower (2002) described a subtype of pathological gamblers with an earlier age of onset and lower insight of illness. A final explanation is that adolescents seem to be less able to perceive gambling as a problem, with a discrepancy between self-perception and the objective problematic behaviors of gambling (Cronce et al., 2007).

Interestingly, QoL resulted is affected by the comorbidity between two or more BAs, especially the mental component. In other words, there is a summative effect of behavioral additions on the mental and physical health of young people.

Logistic regression was performed to ascertain if demographic variables or schema domains were candidates for predicting the occurrence of BAs.

Internet addiction was associated with younger people and almost all EMS domains (with the exception of *impaired autonomy and performance*). Many studies have demonstrated its increasingly young age of onset (Durkee et al., 2012; El Asam et al., 2019). This association could be explained by the self-determination theory where people apply some behaviors in order to satisfy three core needs: competence, autonomy, and relatedness (Deci and Ryan, 2000). In fact, children and adolescents could use the Internet as a coping strategy for depressive feelings (Horwitz et al., 2011) resulting from few physical social interactions, which lead them not to feel emotions and mental states such as shame, shyness, inadequacy, and failure (Chak and Leung, 2004). For this reason, it is not surprising that, in our study, almost all the EMS domains were associated with IA. To our knowledge, our results are in line with the only study that investigated the relationship between EMS and IA (Shajari et al., 2016). It found a significant relationship between the five domains of EMS and IA in students.

Food addiction correlated with the female gender, higher BMI, and the *disconnection and rejection* schema domain. This is in line with the only study that investigated the relationship between EMSs and FAs (Imperatori et al., 2017). The authors found that the *disconnection and rejection* schema was associated with FA, suggesting that this domain may play a key role for the development of FA. Additionally, several studies have highlighted how FA positively correlated with the female gender (Aloi et al., 2017; Carr et al., 2017; Rogers, 2017) and BMI (Aloi et al., 2017; Granero et al., 2018). In fact, a higher prevalence of FA was found among obese patients (Gearhardt et al., 2014).

The occurrence of GD was associated with the male gender and with the *impaired autonomy and performance* domain. To our knowledge, only one study has investigated the relationship between potential GD and EMSs in a sample of alcohol-dependent men (Shorey et al., 2012). Shorey et al. (2012) found that many EMSs were associated with gambling – more specifically, the *insufficient self-control* schema was one of the most significant schema among gamblers. This is in line with our results. In fact, the *insufficient self-control* schema falls within the domain of *impaired autonomy and performance*. Concerning the association between the male gender and pathological gambling, several studies have yielded solid results showing how the male gender was more likely to be associated with GD (Potenza et al., 2002; Villella et al., 2011; Di Nicola et al., 2017).

From the results of the regression analysis, we might think that EMSs could play an important role not only in the development, but also in the maintenance, of BAs. In fact, if by definition, the EMSs are stable and perpetuated over time, when patients with BAs experience emotional triggers, they will reactivate specific EMSs, and this will continue the vicious circle. Just as we said in the introduction, the EMSs are “comprised of memories, emotions, cognitions and bodily sensations regarding oneself and one’s relationship with others,” it could be assumed that many alterations in cognition, in self-perceptions of emotions (i.e., alexithymia), and in the experience of pleasure (i.e., anhedonia), repeatedly reported in the literature among patients with BAs (Guillot et al., 2016; Moccia et al., 2017, 2018), may be associated with the development and perpetuation of EMSs.

### Limitations

Although this study has many strengths (i.e., large sample size, assessment of EMSs in a young population with and without Bas, according to the new model proposed by Bach et al., 2018), some limitations must be acknowledged. First, some BAs, such as compulsive buying or compulsive sexual behavior, were not investigated in our research. Second, this is a cross-sectional study, so our conclusions could just infer an association and not causation. Third, the results cannot be generalized to clinical samples because ours was a sample from the general population. Fourth, the presence or absence of BAs was carried out on the basis of self-reported questionnaires, but these data were not confirmed with face-to-face interviews; nevertheless, in order to reduce the possible high rate of false positive participants, we have used the extreme cut-off values of measures to diagnose each BA. Finally, the participants’ personality features were not evaluated and it is known that addictive disorders are often comorbid with personality disorders (i.e., borderline personality disorders) (Brown et al., 2015).

## Conclusion

Our findings partially confirmed our hypotheses. In fact, mental and physical health perceptions were impaired in IAs and FAs, but not in GDs. Nevertheless, the most striking result is that FA appears to be associated with the *disconnection and rejection* schema domain, IA with all the schema domains (except for *impaired autonomy and performance*), and GD with *impaired autonomy and performance* schema domain. For this reason, EMSs should be systematically assessed within psychotherapy of patients with BAs.

*A priori*, in line with the concept of personalized and precision medicine, ST could provide a real tailor-made therapy addressing the EMSs that seem to underpin the addictive behavior.

## Data Availability Statement

The datasets generated for this study are available on request to the corresponding author.

## Ethics Statement

The studies involving human participants were reviewed and approved by the Ethical Committee of Azienda Ospedaliera Universitaria Mater Domini. Written informed consent to participate in this study was provided by the participants’ legal guardian/next of kin.

## Author Contributions

CS-G and PD contributed to the conception and design of the study. MA, MR, and RS organized the database. MA and CS-G performed the statistical analysis. MA wrote the first draft of the manuscript. MR and VV wrote sections of the manuscript. CS-G, PD, SJ-M, and FF-A critically revised the manuscript. All authors contributed to manuscript revision, and read and approved the submitted version.

## Conflict of Interest

The authors declare that the research was conducted in the absence of any commercial or financial relationships that could be construed as a potential conflict of interest.

## Footnotes

1 https://static-content.springer.com/esm/art%3A10.1007%2Fs40519-017-0421-x/MediaObjects/40519_2017_421_MOESM1_ESM.docx
